# What Makes Consumers’ Intention to Purchase Paid Stickers in Personal Messenger? The Role of Personality and Motivational Factors

**DOI:** 10.3389/fpsyg.2021.678803

**Published:** 2022-01-10

**Authors:** Hyunmin Kang, YounJung Park, Yonghwan Shin, Hobin Choi, Sungtae Kim

**Affiliations:** ^1^Able Edutech, Seoul, South Korea; ^2^Laboratory of Cognitive Engineering, Graduate Program in Cognitive Science, Yonsei University, Seoul, South Korea; ^3^Laboratory of Industrial/Organizational Psychology, Department of Psychology, Yonsei University, Seoul, South Korea; ^4^Laboratory of Cognitive Engineering, Department of Psychology, Yonsei University, Seoul, South Korea

**Keywords:** personality traits, motivations, technology acceptance model, purchase intention, personal messenger, stickers

## Abstract

Many messengers and social networking services (SNSs) use emojis and stickers as a means of communication. Stickers express individual emotions well, allowing long texts to be replaced with small pictures. As the use of stickers increased, stickers were commercialized on a few platforms and showed remarkable growth as people bought and used stickers with their favorite characters, products, or entertainers online. Depending on their personality, individuals have different motivations for using stickers that determine the usefulness and enjoyment of stickers, affecting their purchase decisions. In the present study, participants (*n* = 302) who were randomly recruited from a university completed an online questionnaire assessing the Big Five personality characteristics, motivations for using stickers, and the technology acceptance model (TAM). Results using partial least squares structural equation modeling (PLS-SEM) revealed that each personality trait affected different motivations for using stickers. Moreover, motivations for using stickers also influenced different technology acceptance variables. Finally, perceived usefulness, enjoyment, and ease of use had a positive effect on the intention to purchase stickers. This study has implications in that it is an exploratory approach to the intention to purchase stickers, which has been investigated by few prior studies, and it sheds light on the relationship between personality, motivation, and TAM in purchasing stickers. It also suggests that personality and motivation factors can be considered in personalized recommendation services.

## Introduction

**Figure 1:**
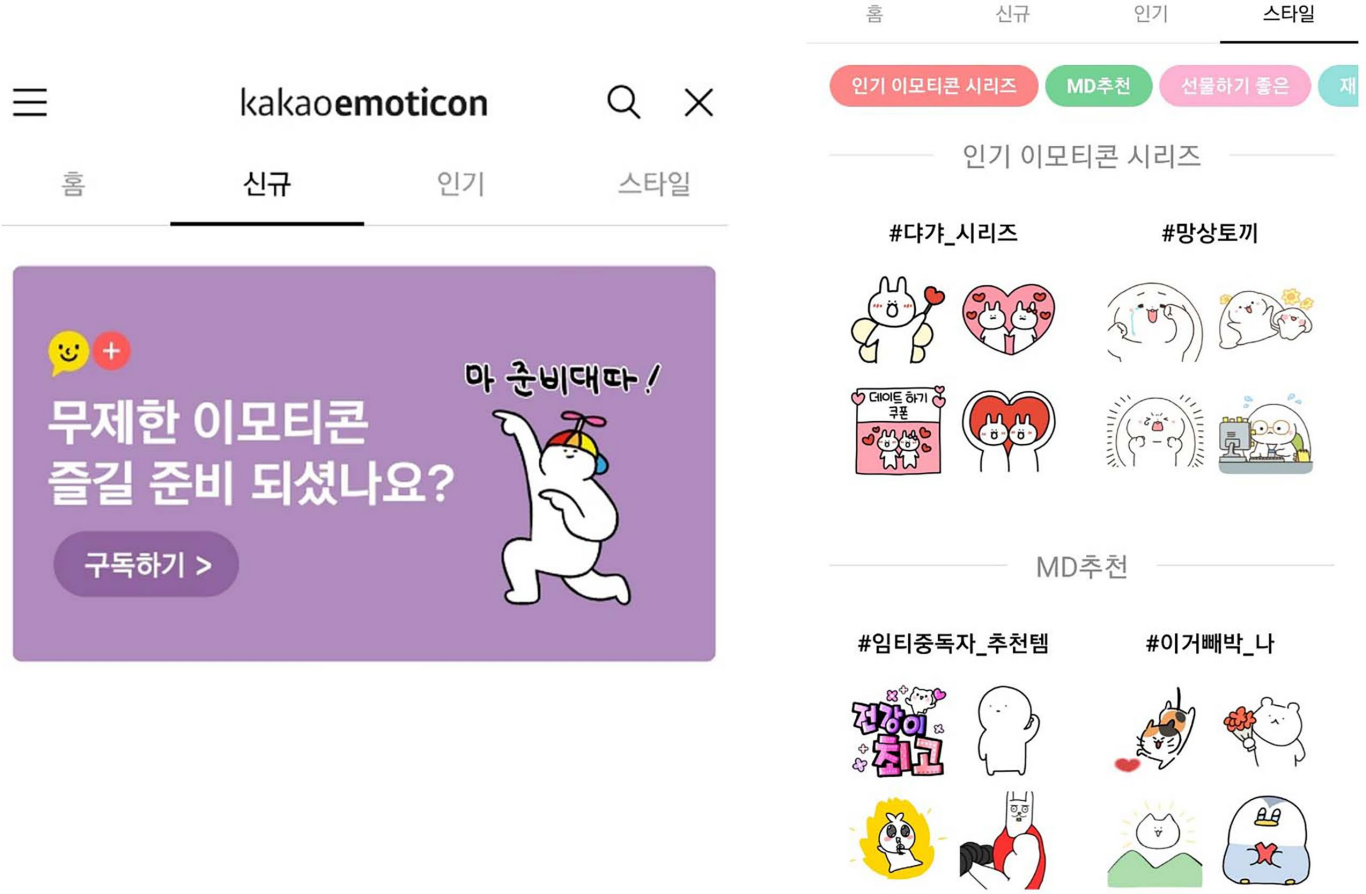
Kakao emoticon shop and example of stickers. Images reproduced with permission of Kakao.

Stickers are developed from emoticons and emojis and are one of the tools that help improve conversations. Emoticons, emojis, and stickers make online conversations and communication more dynamic by increasing social preferences and richness (Aldunate and González-Ibáñez, 2016). Although face-to-face communication is limited online, they can help express emotions or convey meanings and compensate for the absence of non-verbal cues (Walther and D’Addario, 2001; Derks et al., 2008). The earliest emoticons were created by simply combining keyboard characters (e.g., :D, :O). Later, emoticons in the form of icons appeared (e.g., ☺, ☹), and they were called emojis. Recently, character emoticons and moving emoticons have appeared (Figure 1), and they are called stickers. Stickers can help people dynamically express their emotions, opinions, and intentions (Lim, 2015). Stickers are used in personal messengers, on social networking services (SNSs), and for advertisements. A personal messenger allows conversations with an acquaintance or a group, while an SNS allows communication with an unspecified number of people, not just acquaintances. In this context, stickers are mainly used as a tool for communication with people. Moreover, many companies use stickers to promote their products to consumers (Das et al., 2019). They create their own brand symbols with stickers, draw people’s interest, and encourage users to become familiar with their brands. Thus, depending on the domain where the sticker is used, the purpose of using stickers may be different in each case. In this study, we focused on the context of individuals’ use of stickers in communicating with people close to them *via* personal messengers.

There are many personal messenger applications, such as WhatsApp (the most popular messenger in the world), Facebook Messenger, Skype, Telegram, Line, BBM (Blackberry Messenger), QQ Messenger, WeChat, and KakaoTalk. Most messengers offer emoticons, emojis, and stickers as a free option and thus rarely support the emoji or sticker market. However, KakaoTalk, released by Kakao in Korea, has activated a sticker market (named Kakao Emoticon Shop) which people can buy and present stickers of their favorite characters or celebrities. In addition, the character first made for stickers has been used as a model for real products, such as a cup, doll, and wallet. A total of 1,300 stickers has achieved sales of over $100,000, 73 of which have accumulated sales of about $1 million (Sedaily, 2020). Since 2021, Kakao has provided a subscription service, allowing all stickers to be used for a certain amount of monthly payment. The sticker market has been expanding, and there is a sticker recommendation function in the subscription service. It is likely that various factors affect the purchase of stickers. However, few studies have identified the factors related to the purchase of stickers. Therefore, it is meaningful to look at the many different factors that affect the purchase of stickers.

Personality is one of the factors that shows people’s personal differences and is often addressed in relation to service and product purchase and preference. There are many factors that affect people’s personal differences, such as personality, development stage, needs, and attitudes (Evans, 2017), of which the personality traits presented by McCrae and Costa (1999) are more stable over time and across situations than other factors. Therefore, investigating the differences in specific service usage patterns or preferences depending on nature will help with the most permanent and stable personalization. Devaraj et al. (2008) reported that personality is often used in management and psychology research to predict attitudes and behaviors and that personality is also a useful predictor of user attitudes and beliefs in information system research. In previous studies, research on the use and purchase of products and services related to personality has been actively conducted, for instance, M-shopping (Moslehpour et al., 2018; Lissitsa and Kol, 2021), intent to purchase organic food (Chaturvedi et al., 2020), intention to provide an online review (Picazo-Vela et al., 2010), and intention to use tables (Camadan et al., 2018). People buy certain products or services for a certain purpose. In the case of stickers, people purchase them to be used in personal messengers, and this is closely related to the motivation for using them.

Recently, many services have become increasingly personalized, and there are many recommendation systems tailored to people’s personal characteristics. Therefore, research on recommending algorithms has been conducted (Modarresi, 2016; Gorgoglione et al., 2019), as well as on how to reflect individual characteristics (Wu et al., 2018; Jeong et al., 2020). Uhlmann and Lugmayr (2008) predicted users’ personalities according to the way they wrote e-mails, while Peltonen et al. (2020) predicted personalities based on the use of applications on smartphones. Evans (2017) suggested that there is a schema of preferred genres of advertising and advertising music depending on personality. For example, more extraverted people preferred scuba gear advertisements, while those with greater neuroticism rejected them. More introverted people preferred classical music for advertisements, while more extraverted people preferred rap and blues. This personality matching may provide useful information when attempting to provide services tailored to an individual’s personality.

Moreover, some studies were conducted on the personal characteristics associated with the use of stickers. Bai et al. (2019) reviewed studies related to emojis and stickers, among which psychological studies focused on the relationship between user personality traits and emoji use. For example, Li et al. (2018) suggested that negative emojis are negatively related to emotional stability, and positive emojis are positively related to extraversion. Recently, Liu and Sun (2020) studied the correlation between personality types and the reasons for using stickers and found that each personality type was differently correlated with the reasons for using stickers. The results are very interesting because if the reason for using emojis and stickers varies depending on personality and affects the intention to purchase, presenting stickers that suit people’s motivation for using them can positively impact purchase decisions. The reason for using emoticons, emojis, and stickers can eventually be explained by the motivation for use, and we want to first look at the relationship between personality and the motivation for using emoticons based on research of Liu and Sun (2020).

In addition, we want to hypothesize the entire research model by applying a model that can explain the purchase of stickers. We attempted to explain the intention to purchase stickers using the technology acceptance model (TAM). The TAM has been widely used as a model that predicts users’ intent to use, accept, and purchase, such as using SNSs and stickers beyond technologies and services. The TAM was first created as a model for predicting technology acceptance through people’s motivational variables and ease of use, and later, it has evolved into a model for predicting purchase intentions and usage intentions in numerous marketing studies. The important thing is that personality factors and the TAM also seem to be related. Devaraj et al. (2008) used the TAM to connect personality to information system research, and subsequent studies also reported that the Big Five personality types affected the sub-factors of the TAM (Svendsen et al., 2013; Camadan et al., 2018). In this study, we examined the relationship between personality type, motivation, and the TAM. Specifically, we hypothesized that the motivation for using stickers would be related to variables in the TAM, since the TAM considered two variables in model (perceived usefulness and perceived enjoyment) as motivational factors, and these two variables predict variables related to acceptance or intention along with perceived ease of use (PEU). In summary, we examined the relevance of personality to motivation and their effect on the intention to purchase stickers by applying the TAM.

## Literature Review

### Motivations for Using Stickers

The use of stickers in personal messengers is common. Some studies have explained the advantages of using stickers, as mentioned above. However, few studies have explored individuals’ motivations for using stickers. Several studies have measured extrinsic motivation through perceived usefulness, but this is an indirect motivation, as every product or service has specific motivations for use. We introduced several studies that investigated motivations for using emojis and stickers and summarized how we define motivations for using stickers in this study (Table 1). Bai et al. (2019) reviewed studies on emoticons, emojis, and stickers and divided them into two motivation factors (convenience and conduciveness to emotional expression) for using emojis. Tang and Hew (2018) put together a number of studies and divided the motivations for using emojis and stickers into four categories. They proposed that motivations for using emojis are (1) expressing emotions, (2) avoiding misunderstanding and substituting textual expressions, (3) enjoyment and fun, and (4) social purposes. Liu and Sun (2020) measured the motivation for using emojis and stickers through six single questions that assessed (1) expressing emotions, (2) clarifying/disambiguating messages, (3) lightening up the mood, (4) showing a sense of humor, (5) avoiding awkwardness, and (6) ending the conversation. Moreover, there are two studies that explored the motivation for using stickers through a survey methodology. First et al. Chen and Siu (2017) explored four dimensions related to the use of emoticons by Chinese youth: accuracy, sociability, efficiency, and enjoyment. Second, Lee (2017) investigated various emoticon motivations through a survey on 138 Korean students and examined the motivation for emoticon use under four categories determined through exploratory factor analysis (EFA): (1) emotional expression, (2) intimate representation, (3) image management, and (4) sentence complements.

**Table 1:** Previous studies on the motivation for using emojis and stickers.

No.	Authors	Methodology	Factors
1	Bai et al., 2019	Review papers	Convenience
			Conduciveness to emotional expression
2	Tang and Hew, 2018	Review papers	To express emotion
			To avoid misunderstanding and substitute textual expressions
			For enjoyment and fun
			For social purposes
3	Liu and Sun, 2020	Self-production	Express emotions
			Clarify/disambiguate messages
			Lighten up the mood
			Show a sense of humor
			Avoid awkwardness
			End conversation
4	Chen and Siu, 2017	Interview	Accuracy
			Sociability
			Efficiency
			Enjoyment
5	Lee, 2017	Interview Exploratory factor analysis	Emotional expression
			Intimate representation
			Image management
			Sentence complements

Studies related to the motivation for using stickers seem to have common characteristics, both functionally and emotionally. We used a questionnaire based on motivation of Lee (2017) classification and added other motivations that she did not include but suggested in other literatures. Next, we first describe the relationship between personality and motivation and then explain how it relates to the TAM.

### The Big Five Personality Traits

Personality traits represent the characteristics of a person that account for their consistent pattern of behavior (Cervone and Pervin, 2015). Personality domains are stable over time and across situations (Costa and McCrae, 1999). The personality classification method most widely used by psychologists is the Big Five personality traits, under which a person’s personality is categorized into five factors: neuroticism, extraversion, openness, agreeableness, and conscientiousness. Different studies have suggested five personality factors under different names; in this study, we used the names presented by McCrae and Costa (2003): neuroticism, extraversion, openness to experience, agreeableness, and conscientiousness. We summarized each personality trait and explored the relationship between personality and motivation for using stickers.

#### Neuroticism

Neuroticism is characterized by anxiety, self-consciousness, hostility, and mental disease (Devaraj et al., 2008; Tackett and Lahey, 2017). The results of studies on neuroticism differ by domain. First, people with high neuroticism appear to have a negative attitude toward the usefulness of technology. Devaraj et al. (2008) stated that people with high neuroticism consider technical advancement as being threatening and stressful, and thus, neuroticism shows a negative relationship with the perceived usefulness of technology. Jacques et al. (2009) showed that neuroticism is positively correlated with technology communication anxiety in a virtual reality team environment such that a person with high neuroticism worries about future events and responsibilities. In addition, the use of new technology or familiar technology in new ways presents situations in which future outcomes are uncertain.

However, they show a different pattern in online activities, such as SNSs or shopping, rather than in the context of experiencing new technologies or using technologies that require cooperation. McElroy et al. (2007) argued that people with high neuroticism seek information, socialize, and sell products online to escape the stress of face-to-face interaction. Hamburger and Ben-Artzi (2000) noted that neuroticism is positively related to the use of social services. Guadagno et al. (2008) state that people with high neuroticism tend to become bloggers to express themselves online. In summary, people with high neuroticism do not seem to reject new services or technology itself. This depends on what they can do with them. Wang et al. (2012) predicted that people with high neuroticism would show a negative correlation with the perceived usefulness of the use of instant messages but found no such negative correlation with actual use. In other words, neuroticism does not affect the use of the messenger itself, and this allows us to predict that neuroticism will not have a negative effect on the use of stickers in the messenger. Moreover, Li et al. (2018) found that people who score higher on neuroticism more often use emojis and they prefer exaggerated and emotion-rich emojis. Because people with high neuroticism are more likely to experience negative emotions and feelings of stress and anxiety (Yazdani, 2013), they want to express their emotions with emotional emojis. Based on this, we could hypothesize that through emojis or stickers, people with high neuroticism want to express their emotions.

Furthermore, according to Xu et al. (2016), people with high neuroticism tend to use mobile personalization apps. Because they want to improve the quality of existing devices, they are more likely to customize wallpapers, fonts, and ringtones to make their smartphones more unique and attractive. If this holds true for stickers, then people with high neuroticism would be expected to seek to improve the quality of their conversations using emojis or stickers. Therefore, they are likely to use stickers to supplement sentences or convey meaning better.

*H1*: Neuroticism positively affects the motivation for emotional expression.

*H2*: Neuroticism positively affects the motivation for sentence complements.

#### Extraversion

Extraversion is characterized by social, active, outgoing, and warm interpersonal relationships (Watson and Clark, 1997). More extraverted individuals are characterized by energy, dominance, spontaneity, and sociability, whereas more introverted individuals tend to be described as more lethargic, inhibited, reflective, and quiet (Thomas, 2021).

Previous research suggests that people with high extraversion are active in accepting technology. Svendsen et al. (2013) reported that extraversion is positively related to the intention to use a software platform. Camadan et al. (2018) explored the relationship between teachers’ personality and behavioral intention to use tablet PCs and found that extraversion had a positive effect on behavioral intention. Jacques et al. (2009) similarly reported that extraversion predicts perceived usefulness and intention to use a virtual reality team.

However, contradictory results have been reported in the literature. Alabdullatif and Iturbide (2019) showed that extraversion did not affect the use of smart learning technologies, and Barnett et al. (2015) reported that extraversion was negatively related to the actual use of technology, which provides learning information to students. They discussed this result based on the characteristics of extraversion. Extraversion focuses on external sources; therefore, it involves showing better performance when interacting or performing tasks in collaboration with others. In their study, such subjects were unable to interact with people through the learning system, yielding a negative correlation because it was a passive information technology. According to this point of view, it can be assumed that communicating with others through SNSs or messengers is positively correlated with extraversion. Wang et al. (2012) showed that extraversion has direct effects on the perceived enjoyment of using instant messages, and Liu and Campbell (2017) reported that extraversion is significantly associated with SNS use. Additionally, according to DeYoung (2014), extraverted people prefer thrill-seeking activities and social attention. They are also characterized by low emotional arousal (Eysenck, 1967). Thus, highly extraverted people will not simply use stickers to show intimacy or express emotions. Rather, they like to show themselves in their interactions with another person, so stickers will also be used to show themselves better. Recently, Liu and Sun (2020) reported that extraversion is positively correlated with showing a sense of humor in the context of using stickers. People with high extraversion use stickers to show their image more positively. Therefore, their motivation for using stickers may be related to image management.

*H3*: Extraversion positively affects the motivation for image management.

#### Openness to Experience

Openness to experience (openness) is characterized by curiosity, originality, inquisitiveness, and artistic sensitivity (McCrae and Costa, 1989). People with high openness actively seek out new and varied experiences and values. Studies related to openness have shown different results. In some research, openness seems to be related to the intention to use technologies. For example, openness is a personality trait that influences the intention to use a virtual reality team (Jacques et al., 2009). In addition, Devaraj et al. (2008) found that openness is positively related to the intention to use technology. In contrast, Svendsen et al. (2013) found that openness did not affect the intention to use technology; it only affected PEU. Moreover, Xu et al. (2016) suggested that openness is not associated with the adoption of mobile apps. They asserted that the mobile apps used in their study reduced the level of openness because apps became mainstream with a large number of adopters. A more open person is more likely to take risks to achieve a gain (Lauriola and Levin, 2001). Therefore, if a service or technology is already used by many people, it is no longer a risk-taking situation. Using stickers and messengers is an act that most people perform every day, and openness is expected to not play a motivating role in the use or purchase of stickers. Indeed, studies on emojis and stickers have reported that there is no relationship between openness and usage behavior (Marengo et al., 2017; Li et al., 2018; Liu and Sun, 2020). Particularly, Liu and Sun (2020) explored bivariate correlation results between openness and reasons for using stickers, and there were no significant correlations. However, we intended to use a lot of motivation items than Liu and Sun (2020) and categorized them by four factors. Considering that people with high openness love to experience new technology and service, if there is a sticker that can make them be looked trendier, they are likely to use it. In the previous studies, more openness people have high acceptance and use intentions for technologies that are not popularized. It implies that they seem to want to be more early adopters than others. Therefore, if we consider the aspects of openness and interactions with others, people with high openness may be interested in making their image look trendier. We classified four motivations for using stickers and one of them is related to image management which includes items about “look trendy,” “look sensible.” We could hypothesize that people with high openness are related to using stickers to be looked trendy and sensible.

*H4*: Openness positively affects the motivation for image management.

Extroversion and openness are both expected to influence image management, but for different reasons and sizes. Extraversion has a high interest in the image itself shown to others. So, extraversion was found to be greatly affected by the presence or absence of others, and it was not related to technology without interaction with others. But openness is concerned with the new technology itself and the presence or absence of others will not greatly affect. However, considering interactions with others, people with high openness may feel proud of using trendier technology than others. Based on this, we could predict that the openness trait would have a relationship with the motivation that looks trendier and more sensible in the case of using stickers. However, since openness essentially focuses on new experiences rather than relationships with others, it is expected that openness will have less influence than extraversion even if it is related to image management.

#### Agreeableness

An agreeable personality is described as kind, helpful, considerate, and unselfish toward others (Graziano and Eisenberg, 1997; John and Srivastava, 1999). First, agreeableness is related to relationships with others. Seidman (2013) suggested that agreeable individuals are oriented toward others and have belongingness motivation, so they choose Facebook as one way to fulfill those needs. Her results showed that agreeable individuals are more likely to use Facebook to seek acceptance and maintain connections. In addition, Gvili et al. (2020) found that agreeable users seek social value when using SNSs, so it seems that agreeable people are socially oriented toward helping and cooperating with others. Second, in terms of using technology, many studies have reported a relationship between agreeableness and perceived usefulness (Devaraj et al., 2008; Punnoose, 2012). Devaraj et al. (2008) stated that friendly people are more receptive and collaborative and try to see the positive aspects of new technology when dealing with it. Moreover, if technology fosters collaboration, cooperation, and task accomplishment, it will be most strongly related to agreeableness. Finally, a person with high agreeableness tries to express positive emotions toward others with stickers. Marengo et al. (2017) investigated the relationship between personality and types of emojis and found that agreeableness is correlated with emojis depicting blushing faces, and blushing is a signal promoting positive social interactions. Liu and Sun (2020) also reported that agreeableness is positively correlated with expressing emotions, clarifying/disambiguating messages, lightening up the mood, and showing a sense of humor. Applied in our classification, agreeableness is likely to correlate with each motivation. However, considering the basic characteristics of agreeableness and summarizing previous studies, agreeableness is strongly related to emotional interaction with others.

Therefore, people with high agreeableness will want to engage in more emotional exchanges with others, and stickers are also an appropriate service for such emotional communication. Therefore, it was hypothesized that people with high agreeableness would use stickers to better express their emotions and show more intimacy.

*H5*: Agreeableness positively affects the motivation for emotional expression.

*H6*: Agreeableness positively affects the motivation for intimate representation.

#### Conscientiousness

People with high conscientiousness adhere to norms, self-control, and hard work (McCrae and Costa, 2003). Some studies have examined the relationship between conscientiousness and SNS use (Moore and McElroy, 2012; Seidman, 2013; Chua and Chua, 2017; Liu and Campbell, 2017). Conscientious people are reluctant to expose themselves to Facebook (Seidman, 2013), and highly conscientious people express greater regret over posting inappropriate material on Facebook (Moore and McElroy, 2012). Moreover, conscientiousness is negatively related to SNS activities, and conscientious people have a negative attitude toward Facebook (Chua and Chua, 2017; Liu and Campbell, 2017). In addition, they seek information with economic value when using SNSs (Gvili et al., 2020). People with high conscientiousness are strict with themselves and support learning-related online activities but refuse online leisure activities. When adopting mobile apps, they also reject apps for leisure because they are unproductive or distracting (Xu et al., 2016).

The primary purpose of personal messengers is to communicate, and people use stickers for many reasons. If stickers maximize the productivity of the conversation, people with high conscientiousness will use stickers for that purpose. The sentence complement motivation is related to clear communication and highlight the delivery, and these items are related to maximizing the productivity of the conversation. Therefore, we expected that high conscientiousness would be positively associated with motivation for sentence complements.

*H7*: Conscientiousness positively affects the motivation for sentence complements.

We expected that neuroticism and conscientiousness would affect sentence complementation together. However, the process of influencing is expected to be different. As mentioned above, conscientiousness will not use unnecessary stickers, and if they used stickers, its reason would be to complement the sentence. If the use of stickers helps communication even in the slightest, they will take advantage of it. However, the process of affecting sentence complements by neuroticism is a little different. Neuroticism is related to negative emotions, anxiety, and obsessive tendency (Devaraj et al., 2008; Tackett and Lahey, 2017), and this personality trait may be associated with obsessive sticker use patterns. While the use of stickers may serve to complement the text, it will not completely satisfy their needs associated with neuroticism. Therefore, we expected that the explanatory power of obsessive neuroticism affecting sentence complements is less than conscientiousness.

### The Technology Acceptance Model

The TAM began with an interest in the factors that affect information technology when people accept or reject it. Davis (1989) argued that perceived use and PEU affect people’s acceptance. He defined perceived usefulness (PU) as “the degree to which a person believes that using a particular system would enhance his or her job performance” (p. 320) and referred to PEU as “the degree to which a person believes that using a particular system would be free of effort” (p. 320). In other words, PU relates to how helpful this system is, and PEU relates to how easy it is to use. PU and PEU influence behavioral intention, an indicator that determines the actual system use. The TAM has become one of the most widely used models for predicting people’s behavior with respect to technology. For example, Gefen and Straub (1997) studied gender differences by applying the TAM in situations where e-mail was perceived and used, while Park et al. (2013) applied it to smartphone use. It has also been used as a prediction model for people’s acceptance of SNSs (Kwon and Wen, 2010; Rauniar et al., 2014; Mylonopoulos and Theoharakis, 2020). Many consumer research studies have predicted users’ acceptance using the theory of planned behavior (TPB; Ajzen, 1985). As an extended model of the theory of reasoned action (TRA; Ajzen and Fishbein, 1980), the TPB argues that attitudes, social norms, and perceived self-control influence behavioral intentions and lead to actual behavior. Recently, the TAM has been widely used to investigate the acceptance intention, purchase intention (PI), and usage intention of a particular service or SNS (Ramírez-Correa et al., 2019; Holdack et al., 2020; Joe et al., 2020), and some studies have examined the relevance of individual personality traits (Svendsen et al., 2013; Camadan et al., 2018; Harb and Alhayajneh, 2019).

The TAM has been studied with respect to motivation. Davis et al. (1992) described PU as a type of extrinsic motivation and presented an integrated model of technology acceptance that included intrinsic motivation through enjoyment. Later, studies using perceived enjoyment as a tool to measure intrinsic motivation (Agrifoglio et al., 2012; Ramírez-Correa et al., 2019) found that extrinsic motivation and intrinsic motivation both influence continued Twitter usage and SNS usage. Perceived enjoyment (PE) is defined as the extent to which the activity of using the computer is perceived to be enjoyable, apart from any performance consequences that may be anticipated (Davis and Wiedenbeck, 2001). There is one aspect to consider here. Many studies have attempted to use the TAM to explain the motivational aspect, but the questions asked in these studies deal with abstract dimensions. In the case of PU, they generally ask questions related to how using technology improves their performance, productivity, and effectiveness. In addition, in the case of PE, questions measure how interesting contents are. This may be an extrinsic and intrinsic motivation, but it must be expressed more specifically according to the characteristics of each technology. Research that deals with the motivational aspect probes causes one step further. For example, Zheng et al. (2015) carried out an interview study on the motivation for using massive open online courses (MOOCs) and categorized the motivation factors for using MOOCs into fulfilling current needs, preparing for the future, satisfying curiosity, and connecting with people. Moreover, they explored the specificity of the educational environment and the motivations associated with the environmental factors online, suggesting specific motivations that are more contextual than general. The context of using stickers in personal messengers also has particular characteristics. They are mostly online, easy to use, and convenient. This is a common feature of SNSs. However, personal messengers also have distinct characteristics. Lee (2017) examined messenger users through in-depth interviews and found that they mostly used them to communicate with same-gender friends (42.8%), lovers (39.1%), and opposite-gender friends (18.1%). The main conversation partner in a messenger is not an unspecified majority, but a person with whom one has high intimacy. Therefore, it is important to find specific motivations for using stickers in personal messengers and to see how these motivations relate to perceived usefulness and perceived enjoyment.

We have classified the motivations for using stickers into the four factors mentioned previously and hypothesized that these motivations will affect the PU and PE, allowing us to predict people’s intention to purchase stickers. In other words, we present a flow in which people have four motivations for using stickers that affect their perceived usefulness and enjoyment.

*H8*: Motivation for emotional expression positively affects perceived usefulness.

*H9*: Motivation for intimate representation positively affects perceived usefulness.

*H10*: Motivation for image management positively affects perceived usefulness.

*H11*: Motivation for sentence improvement positively affects perceived usefulness.

*H12*: Motivation for emotional expression positively affects perceived enjoyment.

*H13*: Motivation for intimate representation positively affects perceived enjoyment.

*H14*: Motivation for image management positively affects perceived enjoyment.

*H15*: Motivation for sentence improvement positively affects perceived enjoyment.

Finally, the following hypotheses can be established for PU, PE, PEU, and PI.

*H16*: Perceived usefulness positively affects purchase intention.

*H17*: Perceived enjoyment positively affects purchase intention.

*H18*: Perceived ease of use positively affects purchase intention.

## Materials and Methods

**Figure 2:**
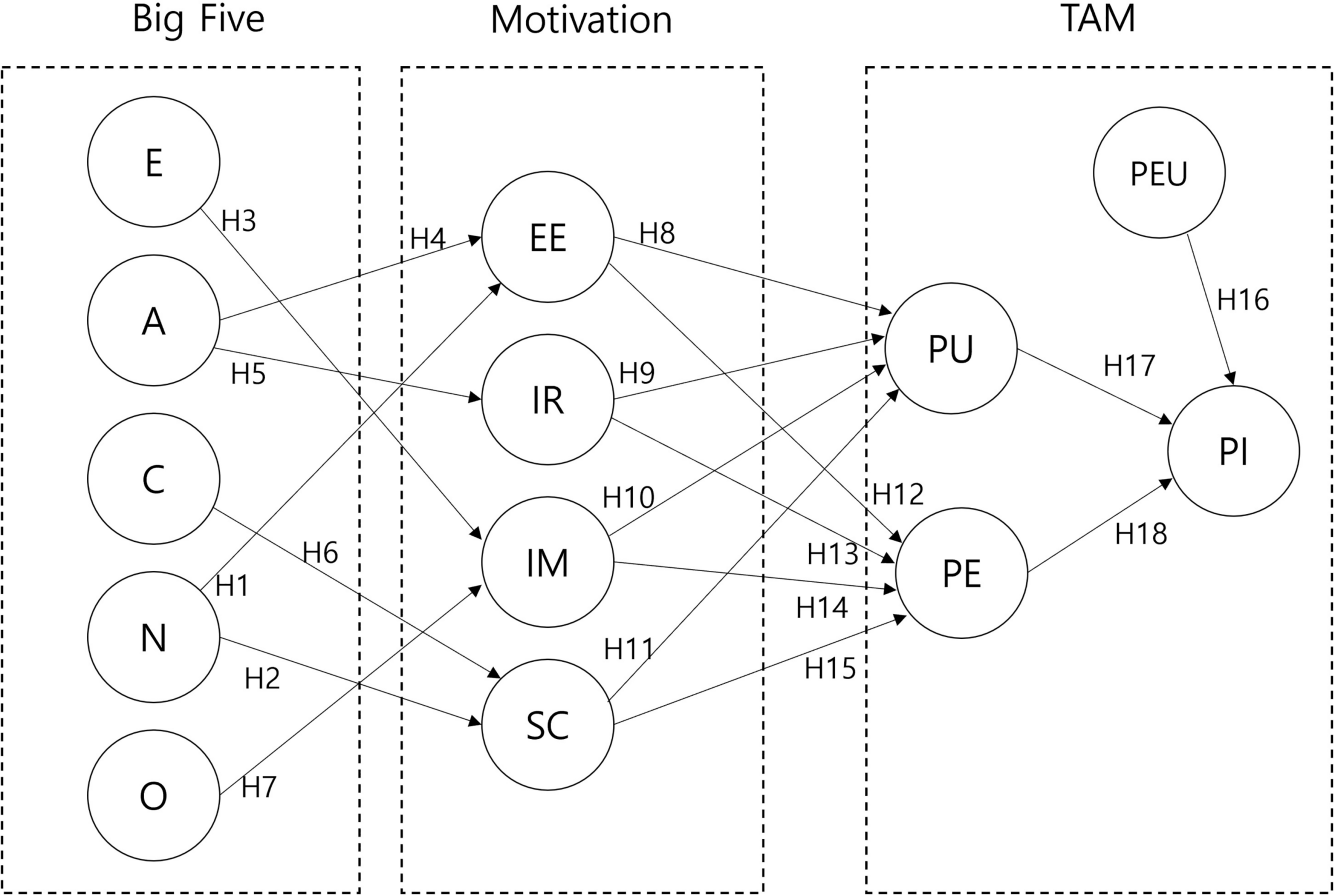
Research model.

We hypothesized the model shown in Figure 2 and conducted a survey to test it. Because it had an exploratory tendency and contained many variables, we tried to verify the model through a PLS-based structural equation, which would not be significantly affected by the number of variables.

### Participants

The participants included 302 university students (198 women and 104 men) with an average age of 25.10 years (SD = 3.25). Participants were students taking psychology classes at the university, and they had more than one experience of purchasing stickers. All the participants were given class credits as compensation for participating in the experiment. The demographic characteristics of the participants are shown in Table 2. Ethical approval was obtained from the Public Institutional Review Board designated by the Ministry of Health and Welfare in Korea (P01-202107-22-005).

**Table 2:** Demographics of participants.

Measures		*N*	%
Gender	Male	104	34.4
	Female	198	65.6
Frequency of daily use	1–10	132	43.7
	11–20	94	31.1
	21–30	50	16.6
	31–40	12	4.0
	>40	14	4.6
Frequency of purchase	1–5	10	3.3
	6–10	148	49.0
	11–15	88	29.1
	>15	56	18.6
Frequency of gift	1–5	70	23.2
	6–10	166	55.0
	11–15	52	17.2
	>15	14	4.6
Number of purchased emoticons	1–5	78	25.8
	6–10	80	26.5
	10–15	34	13.3
	16–20	40	13.2
	>21	70	23.2
Frequency of visiting an emoticon shop	Once 2 weeks	211	69.9
	Once in a month	75	24.8
	Once in 2 months	13	4.3
	Rarely visited	3	1.0

### Questionnaire

To measure the motivation for using stickers, the questionnaire used by Lee (2017) was selected, which categorized motivation into four factors measured using 13 items. Also, we used other seven items from Liu and Sun (2020). We used these 20 questions to measure the motivation for using stickers. Next, we used the 50-item International Personality Item Pool (IPIP) representation of Goldberg (1992) markers for the Big Five factor structure. This tool measures the five personality traits of extraversion (10 questions), agreeableness (10 questions), conscientiousness (10 questions), neuroticism (10 questions), and openness to experience (10 questions). The TAM questionnaire consisted of 12 questions, comprising three questions measuring perceived usefulness (Punnoose, 2012), three questions measuring PEU (Svendsen et al., 2013), three questions measuring perceived enjoyment (Holdack et al., 2020), and three questions measuring purchase intention (Martins et al., 2019). Each question was scored on a 5-point Likert scale ranging from 1 point (*very inaccurate*) to 5 points (*very accurate*). The survey questions are summarized in Table 3. Each factor was tested for reliability and validity through an EFA. In addition, seven questions were asked about gender, age, frequency of daily use, frequency of purchasing emoticons, frequency of purchasing emoticons for gifts, number of purchased emoticons, and frequency of visiting emoticon shop (The results of the frequency analysis for these questions are shown in Table 2). In addition, we used the NASA-TLX workload questionnaire as a marker variable. This questionnaire is composed to six items, and we used three items in it (mental demand, physical demand, and temporal demand). This is because another three items (performance, efforts, and frustration) are not fit in our survey flow, and we need to make our survey simple. Finally, the total number of questionnaire items was 92. The survey was produced and distributed using Google Survey. In the survey, questions were asked using emoticons rather than stickers. This is because emoticons and stickers are not yet distinguished in everyday life, and stickers are mostly called emoticons.

**Table 3:** Questionnaires.

	No.	Items
Extraversion	E1	I am the life of the party.
	E2	I do not talk a lot.
	E3	I feel comfortable around people.
	E4	I keep in the background.
	E5	I start conversations.
	E6	I have little to say.
	E7	I talk to a lot of different people at parties.
	E8	I do not like to draw attention to myself.
	E9	I do not mind being the center of attention.
	E10	I am quiet around strangers.
Agreeableness	A1	I feel little concern for others.
	A2	I am interested in people.
	A3	I insult people.
	A4	I sympathize with others’ feelings.
	A5	I am not interested in other people’s problems.
	A6	I have a soft heart.
	A7	I am not really interested in others.
	A8	I take time out for others.
	A9	I feel others’ emotions.
	A10	I make people feel at ease.
Conscientiousness	C1	I am always prepared.
	C2	I leave my belongings around.
	C3	I pay attention to details.
	C4	I make a mess of things.
	C5	I get chores done right away.
	C6	I often forget to put things back in their proper place.
	C7	I like order.
	C8	I shirk my duties.
	C9	I follow a schedule.
	C10	I am exacting in my work.
Neuroticism	N1	I get stressed out easily.
	N2	I am relaxed most of the time.
	N3	I worry about things.
	N4	I seldom feel blue.
	N5	I am easily disturbed.
	N6	I get upset easily.
	N7	I change my mood a lot.
	N8	I have frequent mood swings.
	N9	I get irritated easily.
	N10	I often feel blue.
Openness to experience	O1	I have a rich vocabulary.
	O2	I have difficulty understanding abstract ideas.
	O3	I have a vivid imagination.
	O4	I am not interested in abstract ideas.
	O5	I have excellent ideas.
	O6	I do not have a good imagination.
	O7	I am quick to understand things.
	O8	I use difficult words.
	O9	I spend time reflecting on things.
	O10	I am full of ideas.
Emotional expression	EE1	I use emoticons to empathize.
	EE2	I use emoticons to convey my feelings naturally.
	EE3	I use emoticons to show my facial expressions in real life.
	EE4	I use emoticons to convey emotions in abundance.
Intimate representation	IR1	I use emoticons to express intimacy.
	IR2	I use emoticons to express interest.
	IR3	I use emoticons to express affection.
Image management	IM1	I use emoticons to look trendy.
	IM2	I use emoticons to look sensible.
	IM3	I use emoticons to look cute.
	IM4	I use emoticons to show a sense of humor.
Sentence complements	SC1	I use emoticons to highlight the delivery.
	SC2	I use emoticons for clear communication.
	SC3	I use emoticons to complement the text.
	SC4	I use emoticons to clarify messages.
Perceived ease of use	PEU1	Learning to use emoticons is easy for me.
	PEU2	It would be easy for me to become skillful in using emoticons.
	PEU3	My interaction using emoticons is clear and understandable.
Perceived usefulness	PU1	Using emoticons improves my conversation in the messenger.
	PU2	Using emoticons enables me to do my message more efficiently.
	PU3	Using emoticons increases my productivity.
Perceived enjoyment	PE1	Using emoticons makes me feel good.
	PE2	Using emoticons is enjoyable.
	PE3	Using emoticons is interesting.
Purchase intention	PI1	I find purchasing emoticons to be worthwhile.
	PI2	I will frequently purchase emoticons in the future.
	PI3	I will strongly recommend others to purchase emoticons.

#### Procedure

Participants in the experiment were asked to voluntarily participate through the recruitment bulletin board within the university and were given credits in exchange for participation. When they agreed to participate, the questionnaires for motivation, personality, TAM, and personal background were administered in order, which took about 20 min to complete.

## Results

### Exploratory Factor Analysis

First, EFA was used to ensure that each factor had the same structure as previous studies. We used SPSS 26 for analysis, and EFA was based on the suggestion of Fabrigar et al. (1999) using the maximum likelihood extraction method and oblique rotation. We deleted items whose factor loadings were less than 0.3 based on method of Floyd and Widaman (1995). Also, any item which cross-loaded on two factors with factor loadings higher than 0.3 was removed. We input all items of the Big Five, motivations for using sticker and TAM sub-factors, and the items were classified into 13 factors. As a result of the analysis, 13 factors could be divided into five personality factors (extraversion, agreeableness, conscientiousness, neuroticism, and openness), four motivation factors (emotional expression, intimate representation, image management, and sentence complement), and four TAM sub-factors (PEU, PU, PE, and PI), respectively (Table 4). Particularly, two items used in Liu and Sun (2020) were included in four categories of Lee (2017), and another five items were deleted because of low factor loadings.

**Table 4:** Cross-loadings of items.

	Items	E	A	C	N	O	EE	IR	IM	SC	PEU	PU	PE	PI
E	E2	0.798	−0.056	−0.014	−0.098	0.040	−0.022	−0.054	−0.011	0.002	0.057	0.040	0.019	0.029
	E3	0.630	−0.055	0.003	0.086	−0.012	0.023	0.088	−0.026	−0.010	−0.044	0.008	−0.005	−0.112
	E4	0.787	0.009	−0.056	−0.083	−0.022	0.005	−0.082	−0.010	0.009	0.088	−0.013	0.051	0.102
	E5	0.706	0.040	−0.020	0.008	−0.055	0.165	0.015	0.108	−0.073	0.069	0.024	−0.010	0.054
	E6	0.643	−0.138	−0.068	0.067	0.040	−0.099	0.047	−0.029	−0.022	−0.090	0.048	−0.012	−0.092
	E7	0.692	−0.011	0.026	0.033	−0.018	0.010	0.071	0.031	0.027	−0.024	0.031	−0.029	−0.114
	E8	0.646	−0.007	−0.005	−0.001	−0.049	−0.029	0.067	−0.048	0.053	0.011	−0.108	0.096	0.070
	E9	0.560	0.042	0.009	0.008	−0.030	0.009	−0.017	−0.005	0.071	0.035	−0.125	0.114	−0.027
	E10	0.674	−0.027	0.010	0.113	−0.072	0.049	−0.126	0.147	−0.081	−0.049	0.104	−0.113	−0.037
A	A1	−0.032	−0.804	−0.055	0.010	−0.095	0.028	−0.122	−0.014	0.070	0.040	−0.033	0.056	0.054
	A2	0.274	−0.356	0.069	−0.071	−0.058	0.016	0.243	−0.029	0.110	−0.073	−0.143	−0.069	−0.194
	A3	−0.063	−0.298	−0.117	0.208	0.051	0.127	−0.013	−0.056	−0.077	0.054	0.153	−0.133	0.021
	A4	0.057	−0.639	0.071	−0.141	0.021	0.068	0.142	0.012	−0.053	−0.007	−0.001	0.007	−0.070
	A6	0.141	−0.513	−0.026	−0.032	−0.052	−0.011	0.213	−0.029	−0.107	−0.032	0.087	0.058	−0.025
	A7	0.264	−0.394	0.023	−0.115	0.035	−0.050	0.117	−0.016	0.089	−0.139	−0.006	−0.098	−0.182
	A9	−0.001	−0.765	0.038	−0.004	−0.030	−0.005	−0.086	0.083	0.058	0.110	−0.034	0.113	0.042
C	C1	−0.018	−0.029	−0.643	−0.015	−0.062	0.069	0.144	−0.061	0.013	−0.029	0.067	0.023	0.016
	C2	0.024	0.059	−0.904	0.000	0.024	−0.062	−0.034	−0.001	0.065	−0.021	−0.036	−0.040	−0.050
	C3	0.004	−0.175	−0.485	−0.115	−0.141	0.012	0.067	−0.014	0.050	−0.023	0.124	−0.027	0.046
	C4	0.016	−0.011	−0.895	0.048	0.020	−0.018	−0.077	0.037	0.007	−0.054	−0.037	−0.011	−0.032
	C5	0.045	0.048	−0.498	0.047	−0.020	0.069	−0.031	−0.077	−0.018	0.028	0.063	−0.004	−0.108
	C6	−0.041	−0.068	−0.771	0.024	0.070	−0.075	−0.094	0.090	0.061	0.048	−0.098	−0.041	0.007
	C7	0.060	0.104	−0.723	−0.069	0.026	−0.008	0.105	0.018	−0.037	−0.004	0.008	0.123	0.137
N	N1	−0.096	−0.038	−0.057	−0.737	0.022	0.107	0.034	0.032	−0.114	−0.019	0.090	−0.044	0.002
	N2	−0.154	0.054	−0.119	−0.476	−0.046	0.077	−0.007	−0.043	−0.073	−0.030	0.041	−0.073	−0.126
	N5	−0.071	−0.026	0.055	−0.775	−0.032	0.119	0.018	−0.034	−0.123	−0.137	0.048	0.020	0.026
	N6	0.051	0.009	−0.080	−0.530	0.008	−0.072	−0.140	0.036	0.148	0.017	−0.017	0.039	−0.038
	N7	0.135	−0.094	0.079	−0.838	0.103	−0.023	0.039	−0.006	−0.022	0.054	0.033	0.012	0.033
	N8	0.073	−0.068	0.045	−0.872	0.001	−0.065	0.034	−0.015	0.076	0.010	0.027	−0.034	−0.012
	N9	0.019	0.081	−0.013	−0.694	0.056	−0.040	−0.031	0.002	0.103	0.032	−0.065	0.076	0.027
	N10	−0.062	−0.056	0.079	−0.758	−0.060	0.081	0.043	0.032	−0.063	0.002	−0.062	−0.034	0.046
O	O3	−0.069	−0.025	0.141	−0.094	−0.857	0.037	−0.030	0.086	0.008	0.017	−0.011	−0.015	0.047
	O4	0.085	−0.042	−0.131	0.095	−0.402	−0.026	0.036	−0.072	−0.068	0.055	−0.057	−0.001	−0.009
	O5	0.144	0.013	−0.031	0.098	−0.626	−0.075	0.020	0.063	0.044	0.046	0.033	−0.003	0.006
	O6	−0.011	−0.035	−0.028	0.020	−0.851	−0.009	−0.099	−0.029	−0.019	−0.077	0.021	0.016	0.026
	O9	−0.143	−0.026	−0.037	−0.115	−0.577	0.043	0.060	−0.014	−0.023	0.007	−0.007	0.025	−0.052
	O10	0.183	0.047	0.083	0.112	−0.774	−0.079	0.028	0.003	0.101	−0.028	0.041	−0.028	−0.038
EE	EE1	0.092	−0.018	0.015	−0.039	−0.059	0.562	0.066	0.042	0.053	−0.036	−0.008	0.014	−0.016
	EE2	−0.016	−0.035	−0.019	−0.019	0.080	0.744	0.087	−0.039	0.072	0.097	0.007	−0.015	−0.019
	EE3	0.018	0.006	0.026	−0.033	−0.025	0.395	−0.039	0.038	0.156	−0.198	0.061	0.087	−0.078
	EE4	0.020	−0.047	0.016	−0.035	0.125	0.669	0.054	0.035	0.156	0.046	−0.026	0.039	−0.057
IR	IR1	−0.069	−0.028	0.012	0.059	−0.059	0.081	0.488	0.067	0.092	0.097	−0.055	0.109	−0.047
	IR2	−0.049	0.007	−0.053	0.032	0.018	0.010	0.743	0.115	0.052	0.047	−0.007	0.020	−0.043
	IR3	0.011	0.034	0.008	0.009	−0.010	0.075	0.690	−0.014	0.044	0.072	0.053	0.027	0.022
IM	IM1	−0.065	−0.085	0.023	−0.021	−0.010	−0.128	0.001	0.836	0.116	−0.099	−0.017	−0.053	−0.061
	IM2	0.019	0.024	−0.008	0.055	−0.006	0.021	0.071	0.788	−0.051	−0.029	0.017	−0.018	−0.053
	IM3	0.083	0.031	−0.021	−0.102	0.035	0.028	0.286	0.405	−0.021	−0.007	0.073	0.067	0.025
	IM4	0.064	0.005	−0.022	0.021	−0.037	0.172	−0.040	0.385	−0.053	0.057	0.028	0.096	0.004
SC	SC1	0.024	0.038	−0.037	−0.020	−0.090	0.118	0.082	0.003	0.633	−0.070	0.101	0.035	0.040
	SC2	−0.118	−0.074	−0.137	0.071	0.059	0.078	0.059	0.005	0.730	−0.050	0.045	−0.018	−0.069
	SC3	0.025	−0.027	−0.030	−0.022	−0.042	0.175	0.009	0.062	0.533	0.183	0.046	−0.020	0.052
	SC4	0.052	−0.014	0.051	−0.007	0.056	0.090	0.097	−0.020	0.429	−0.021	0.204	−0.018	−0.088
PEU	PEU1	−0.020	−0.039	0.011	−0.059	−0.005	−0.036	0.048	0.003	−0.082	0.540	0.196	0.006	−0.193
	PEU2	−0.001	−0.060	0.041	0.032	0.019	0.001	0.056	−0.024	0.037	0.875	−0.061	0.018	−0.065
	PEU3	0.093	−0.024	−0.004	0.007	−0.036	−0.007	0.081	−0.079	0.028	0.732	0.072	0.008	−0.064
PU	PU1	−0.009	0.070	0.006	−0.003	0.012	0.160	0.080	−0.028	0.096	0.163	0.458	0.231	−0.181
	PU2	−0.006	−0.028	0.007	−0.048	−0.030	−0.053	−0.035	0.118	0.144	0.059	0.769	0.109	−0.012
	PU3	−0.008	0.087	−0.050	−0.028	0.002	−0.006	0.017	−0.011	0.215	0.106	0.653	0.153	−0.066
PE	PE1	0.045	−0.085	0.027	0.023	−0.027	−0.031	0.033	−0.019	−0.007	0.003	0.096	0.781	−0.116
	PE2	−0.010	−0.088	−0.043	0.059	−0.032	0.014	0.035	−0.056	−0.005	−0.011	0.056	0.932	−0.030
	PE3	0.007	−0.033	−0.023	−0.022	0.087	0.084	0.034	0.144	−0.087	0.015	0.049	0.670	−0.138
PI	PI1	0.000	−0.012	−0.013	0.049	−0.040	0.096	−0.016	0.070	−0.036	0.153	−0.034	0.154	−0.633
	PI2	−0.005	0.008	0.014	−0.059	0.022	0.101	0.008	−0.010	−0.014	0.173	0.037	0.131	−0.738
	PI3	0.018	0.063	−0.008	−0.015	−0.017	−0.046	0.033	0.119	0.054	0.053	0.096	0.114	−0.685

E, extraversion; A, agreeableness; C, conscientiousness; N, neuroticism; O, openness to experience; EE, emotional expression; IR, intimate representation; IM, image management; SC, sentence complement; PEU, perceived ease of use; PU, perceived usefulness; PE, perceived enjoyment; PI, purchase intention.

### Tests for Reliability and Validity

To examine the proposed hypotheses, we used SmartPLS 3.3.3 to analyze data using partial least squares (Ringle et al., 2015). The analysis was conducted in two stages to first evaluate the outer model and then the inner model. The outer model evaluation consisted of verifying the reliability and validity. To confirm the reliability and convergent validity of the factors, we checked the values of Cronbach’s alpha, composite reliability (CR), average variance extracted (AVE), and outer loadings. To ensure validity, the CR value must be greater than 0.7 and the AVE must be greater than 0.5. It is desirable that the outer loadings of each item exceed 0.7, and those with values less than 0.4 should be removed. However, if the value is greater than 0.4 and less than 0.7, the item should be removed if the AVE and CR values rise above the threshold when the item is deleted. It is also possible to decide whether to delete an item by considering content validity aspects (Hair et al., 2011). The results showed that the reliability and convergent validity of all factors were suitable for analysis, items with low outer loadings were deleted through these criteria, and the factors were constructed for the remaining items. Next, we checked the Fornell–Larcker criterion and cross-loadings (Table 4) to assess discriminant validity and found that the square root of AVE was higher than the correlation coefficient value for all factors. Finally, we checked the inner variance inflation factors (VIFs) value and found no VIF values over 5. Specific values related to reliability and validity are summarized in Table 5.

**Table 5:** Reliability and convergent validity.

Variables	Items	Outer loadings	VIF	Cronbach’s ***α***	CR	AVE
Extraversion	E2	0.777	2.468	0.897	0.914	0.542
	E3	0.717	1.861			
	E4	0.760	2.286			
	E5	0.808	2.055			
	E6	0.693	1.822			
	E7	0.800	2.120			
	E8	0.668	2.195			
	E9	0.620	1.879			
	E10	0.758	1.908			
Agreeableness	A1	0.672	1.993	0.827	0.870	0.529
	A2	0.766	2.022			
	A4	0.803	1.858			
	A6	0.746	1.585			
	A7	0.701	1.998			
	A9	0.668	2.083			
Conscientiousness	C1	0.845	2.285	0.879	0.901	0.568
	C2	0.805	3.709			
	C3	0.758	1.629			
	C4	0.780	3.701			
	C5	0.636	1.553			
	C6	0.689	2.138			
	C7	0.743	1.910			
Neuroticism	N1	0.810	2.591	0.892	0.915	0.578
	N2	0.571	1.412			
	N5	0.818	2.815			
	N6	0.606	1.746			
	N7	0.831	3.057			
	N8	0.872	3.541			
	N9	0.746	2.184			
	N10	0.772	2.415			
Openness	O3	0.842	2.566	0.852	0.885	0.612
	O5	0.833	1.811			
	O6	0.777	2.637			
	O9	0.530	1.354			
	O10	0.878	2.477			
Emotional expression	EE1	0.767	1.594	0.785	0.861	0.609
	EE2	0.828	1.892			
	EE3	0.664	1.361			
	EE4	0.850	1.953			
Intimate representation	IR1	0.770	1.387	0.764	0.865	0.682
	IR2	0.885	2.061			
	IR3	0.819	1.743			
Image management	IM1	0.736	1.873	0.720	0.824	0.540
	IM2	0.801	1.946			
	IM3	0.731	1.239			
	IM4	0.663	1.171			
Sentence complements	SC1	0.822	1.811	0.783	0.860	0.607
	SC2	0.819	1.756			
	SC3	0.745	1.465			
	SC4	0.726	1.353			
Perceived ease of use	PEU1	0.830	1.550	0.823	0.894	0.739
	PEU2	0.887	2.389			
	PEU3	0.861	2.189			
Perceived usefulness	PU1	0.880	1.876	0.856	0.912	0.775
	PU2	0.871	2.304			
	PU3	0.890	2.440			
Perceived enjoyment	PE1	0.905	2.979	0.892	0.933	0.823
	PE2	0.931	3.631			
	PE3	0.885	2.2174			
Purchase intention	PI1	0.861	2.041	0.858	0.913	0.779
	PI2	0.915	2.556			
	PI3	0.870	2.089			

We collected data at a single point in time using a single survey. Therefore, our data are at risk of common method bias (CMB). We performed Harman’s single-factor test (Podsakoff et al., 2003) and the PLS marker variable analysis suggested by Rönkkö and Ylitalo (2011). In Harman’s single-factor test, we carried out an EFA with principal component analysis and an unrotated factor solution. The result was that a total of 16 factors with an eigenvalue greater than 1.0 were extracted. These factors represented 69.49% of the total variance, and the largest factor explained only 14.88% of the variance. This result indicates that CMB was not a problem in this study. Second, we performed a PLS marker variable analysis. We selected marker variables that were not included in the research model. We used a workload questionnaire including mental demand, physical demand, and temporal demand as a marker variable because it looked like to check participants’ perceived difficulty while they survey, and it did not have an explicit theoretical influence on other constructs. Furthermore, the average point of three workload items showed low correlations with other variables. We input the marker variable as a predictor of all the endogenous latent variables in our model. Finally, we compared the models with and without marker variables and found that the significant paths in the baseline model remained significant in the method factor model (Table 6). As a result, the significance of the path was the same regardless of whether the marker variable was inserted. Thus, we concluded that CMB did not occur in the research model.

**Table 6:** The result of common method bias assessment.

Path	Without marker variable	With marker variable
N → EE	0.14^*^	0.12^*^
N → SC	0.08	0.07
E → IM	0.22^***^	0.22^***^
A → EE	0.24^***^	0.24^***^
A → IR	0.31^***^	0.30^***^
C → SC	0.20^***^	0.21^***^
O → IM	0.05	0.05
EE → PU	0.18^**^	0.18^**^
IR → PU	0.13^*^	0.12^*^
IM → PU	0.10^*^	0.10^*^
SC → PU	0.35^***^	0.35^***^
EE → PE	0.13^*^	0.13^*^
IR → PE	0.21^**^	0.21^**^
IM → PE	0.22^***^	0.22^***^
SC → PE	0.05	0.06
PEU → PI	0.24^***^	0.24^***^
PU → PI	0.25^***^	0.24^***^
PE → PI	0.35^***^	0.35^***^
Marker → EE		0.07
Marker → IR		0.10
Marker → IM		0.02
Marker → SC		0.04
Marker → PU		0.04
Marker → PE		0.04
Marker → PI		0.04

^*^p < 0.05; ^**^p < 0.01; ^***^p < 0.001.

### Analysis of the Structural Model

The analysis results of the inner model are as follows. The explanatory power of the structural model is explained by the squared multiple correlations (*R*^2^) and the significance levels of the path coefficients. For the analysis, we set the maximum iterations to 300 and the stop criterion (10^−x^) to seven. Furthermore, we used a bootstrap procedure with 2,000 resamples. The descriptive statistics and correlation results for the variables are presented in Table 7.

**Table 7:** Descriptive statistics and correlations.

	E	A	C	N	O	EE	IR	IM	SC	PEU	PU	AT	PI	*M*	SD
E	1													3.09	0.77
A	0.42^**^	1												3.67	0.67
C	0.06	0.06	1											3.23	0.83
N	−0.11	0.14^*^	−0.02	1										2.98	0.84
O	0.22^**^	0.20^**^	0.07	−0.04	1									3.38	0.79
EE	0.10	0.23^**^	0.05	0.16^**^	−0.03	1								3.52	0.77
IR	0.15^*^	0.26^**^	0.02	0.02	0.06	0.33^**^	1							4.04	0.68
IM	0.22^**^	0.12^*^	−0.02	0.07	0.08	0.26^**^	0.28^**^	1						2.94	0.86
SC	0.13^*^	0.18^**^	0.18^**^	0.06	0.03	0.43^**^	0.31^**^	0.18^**^	1					3.52	0.83
PEU	0.10	0.12^*^	0.03	−0.08	0.01	0.10	0.25^**^	0.00	0.09	1				4.17	0.64
PU	0.06	0.08	0.17^**^	0.07	0.02	0.38^**^	0.30^**^	0.24^**^	0.49^**^	0.37^**^	1			3.85	0.72
PE	0.22^**^	0.14^*^	0.04	0.06	0.06	0.28^**^	0.31^**^	0.31^**^	0.21^**^	0.29^**^	0.47^**^	1		3.91	0.72
PI	0.18^**^	0.21^**^	0.02	0.04	0.04	0.30^**^	0.33^**^	0.27^**^	0.27^**^	0.41^**^	0.48^**^	0.52^**^	1	3.80	0.71

^*^p < 0.05; ^**^p < 0.01.

Table 8 presents the results for *R*^2^. According to the results, it seems that the influence of personality on motivation is relatively small. In particular, intimate representation (0.06) and sentence complements (0.05) have an explanatory power of less than 0.1. Emotional expression and image management have an explanatory power of 0.10. However, the motivation for using stickers has predictive accuracy in perceived enjoyment (0.20) and perceived usefulness (0.32). Finally, perceived usefulness and enjoyment show considerable predictive accuracy for purchase intention (0.44).

**Table 8:** Squared multiple correlations between endogenous variables.

Endogenous variables	*R* ^2^
Emotional expression	0.10
Intimate representation	0.10
Image management	0.06
Sentence complements	0.05
Perceived usefulness	0.32
Perceived enjoyment	0.20
Purchase intention	0.44

The results of the analysis of the path coefficients are presented in Table 9, and the judgment of each hypothesis is also shown. Hypotheses 2, 7, and 15 were rejected, and all other hypotheses were adopted. Neuroticism had a positive effect on emotional expression (H1) but had no effect on sentence complements (H2). Extraversion had a positive effect on image management (H3), agreeableness positively affected both emotional expression (H4) and intimate representation (H5), and conscientiousness had a positive effect on sentence complements (H6). However, openness had no effect on image managements (H7).

**Table 9:** Path analysis and hypothesis testing results.

Hypothesis	Path	Path coefficient	*t*-value	Results
*H1*	N → EE	0.14	2.29^*^	Supported
*H2*	N → SC	0.08	1.12	Not supported
*H3*	E → IM	0.22	4.34^***^	Supported
*H4*	A → EE	0.24	4.36^***^	Supported
*H5*	A → IR	0.31	5.29^***^	Supported
*H6*	C → SC	0.20	3.61^***^	Supported
*H7*	O → IM	0.05	0.67	Not supported
*H8*	EE → PU	0.18	2.99^**^	Supported
*H9*	IR → PU	0.13	2.16^*^	Supported
*H10*	IM → PU	0.10	1.99^*^	Supported
*H11*	SC → PU	0.35	6.08^***^	Supported
*H12*	EE → PE	0.13	2.10^*^	Supported
*H13*	IR → PE	0.21	3.37^**^	Supported
*H14*	IM → PE	0.22	3.54^***^	Supported
*H15*	SC → PE	0.05	0.90	Not supported
*H16*	PEU → PI	0.24	5.06^***^	Supported
*H17*	PU → PI	0.25	4.40^***^	Supported
*H18*	PE → PI	0.35	6.75^***^	Supported

^*^p < 0.05; ^**^p < 0.01; ^***^p < 0.001.

**Figure 3:**
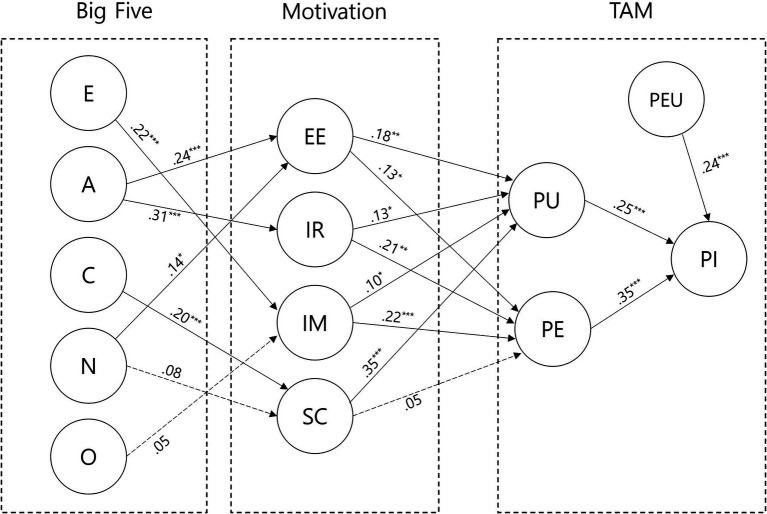
Model results. *p < 0.05; **p < 0.01; ***p < 0.001.

Consequently, it was confirmed that each personality type was related to different motivations. Next, except between sentence complements and PE (H15), every motivation factor affects PU and PE, respectively. Emotional expression positively affected PU (H6) and PE (H12), and intimate representation also positively affected PU (H7) and PE (H13). Image management had a positive effect on PU (H10) and PE (H14). Sentence complements affected only PU (H8). Lastly, PEU (H16), PU (H17), and PE (H18) had a positive effect on purchase intention. The results of the model are presented in detail in Figure 3.

Furthermore, this study is an exploratory research, so we compared our research model to a saturated model that included all possible paths. Gefen et al. (2011) proposed that research verified that (1) the significant paths in the theoretical model also remain significant in the saturated model and (2) adding the paths *via* the saturated model does not significantly increase *f*^2^, a standard measure of effect size. In this study, all hypothetical paths were significant in the saturated model, and the *f*^2^ values of all variables did not increase. We also examined whether there was a difference in the *R*^2^ and *Q*^2^ of purchase intention, the ultimate endogenous variable of the study. As a result, there was almost no difference in *R*_adj_^2^ between the research model and the saturated model (research model: 0.436, saturated model: 0.448), as well as *Q*^2^ (research model: 0.334, saturated model: 0.339).

## Discussion

The purpose of this study was to examine how individual personality and motivational factors affect PU and PE, and PU, PEU, PE affect the intention to purchase stickers. To summarize the results, it appears that the motivation for using stickers varies depending on personality traits. In this study, we classified motivations into four categories, and the results showed that neuroticism is related to emotional expression, extraversion is related to image management and agreeableness is related to motivation for emotional expression and intimate representation. Since conscientiousness values self-control or hard work, as mentioned earlier, people with high conscientiousness are more likely to use stickers for sentence complements. Contrary to expectations, neuroticism does not seem to be related to sentence complement motivation (H2). Xu et al. (2016) carried out a study on using apps on smartphones and found that people with high neuroticism used apps that would make their smartphones more unique. In this study, we expected that these personality features would be related to behaviors that increase the quality of conversation, but the results are different. People with high neuroticism probably prefer uniqueness rather than supplementing the conversation or enhancing its quality. Butt and Phillips (2008) reported that people with high neuroticism try to control information, and Ross et al. (2009) reported that these people prefer Facebook’s Wall service to control information quickly. In this study, we predicted that people with high neuroticism recognized that conversation would be more unique and developed with stickers, based on the previous results that these people tried to make be unique and develop themselves. However, it seems that what is more important to these people is the protection and control of their information, not supplementation of deficiencies in the conversation. Rather, the tendency to fill in the parts of the conversation that are lacking appears to be closer to conscientiousness (H6). Therefore, high neuroticism is not related to the motivation for improving or complementing the quality of the conversation through stickers. In addition, Liu and Sun (2020) reported a positive correlation between neuroticism and awkwardness avoidance and ending a conversation. It has also been reported that there is a negative correlation between neuroticism and showing a sense of humor. Considering findings of Liu and Sun (2020), people with high neuroticism appear to use stickers as a tool to supplement an awkward situation, in other words, in a situation where negative emotions are likely to be induced. Therefore, it seems that the neuroticism trait affected emotional expression (H1). People with high neuroticism are likely to express their negative emotions using stickers. In our survey, emotional expression motivation did not check whether it is a positive emotion or not. If we look at more diverse types of motivations for using stickers in the future, we will reveal what motivations are related to neuroticism.

Second, the openness trait did not affect image management (H7). This result shows that high openness is an interest in new experiences that have not been experienced much. We expected that people with high openness would be interested in comparisons with others, so we hypothesized that openness would have a relationship with managing their image to others. However, this hypothesis is wrong, and openness is thought to be more related to external curiosity, regardless of comparison with others or the image shown to others. So, it seems that this result is the same as previous studies that explored between openness and the use of stickers (Marengo et al., 2017; Li et al., 2018; Liu and Sun, 2020).

Moreover, four motivation factors affect PU and PE. Each motivation to use the sticker is ultimately related to both intrinsic motivation and extrinsic motivation. In other words, the various motivations for using stickers all affect the usefulness and enjoyment of using stickers. Interestingly, sentence complements motivation does not affect PE. It is often explained that PU is related to extrinsic motivation and PE is related to intrinsic motivation. Therefore, PE relates to the hedonic value and describes how the service gives subjective pleasant experience (Holdack et al., 2020). Zhou and Feng (2017) reported that PU has a greater influence when video calling is a work context, and PE has a greater influence when it is in a leisure context. From this point of view, we can predict that motivations that give pleasure to one’s inner self are related to PE and the functional motivation to do something through stickers is related to PU. In this study, people seem to feel all motivations for using stickers are functionally useful. This result is looked obvious, as stickers can perform various functions that were not possible with texts. However, the relationship with intrinsic enjoyment is associated with the emotionally pleasurable factor. Emotional expression, intimate representation, and image management all have to do with expressing one’s emotions or showing a better image, and this seems to affect the inner enjoyment factor. On the contrary, in the case of sentence complements, it seems that this motivation factor is entirely related to functionally supplementing the sentence better. So, we could conclude that this motivation did not affect PE. Finally, similar to the previous research, a relationship was identified whereby PEU, PU, and PE affected purchase intention. The explanatory power of purchase intention is high, suggesting that the purchase intention can be highly predicted by the variables of TAM.

Each personality has a different motivation for using stickers, and motivation differently affects purchase intention through PU and PE, suggesting that each personality might have a different preference for stickers. People with high extraversion may prefer novel cute stickers or trendy characters, and there is a high possibility of choosing a sticker that can show off one’s specific image among many stickers. People with high agreeableness will prefer stickers with more emotional expressions, such as facial expressions and stickers with intimacy. People with high conscientiousness may not prefer cuter or more intimate stickers. Rather, they want to use more informative stickers, such as those conveying the exact message. There are many ambiguities in the interpretation of emojis and stickers. Miller et al. (2016) state that people often interpret the same emoji differently. People with high conscientiousness are not likely to use these kinds of stickers, and they are likely to choose stickers with no uncertainty. Of course, a variety of individual difference factors can affect motivation or purchase intention, but at least in the context in which those factors are controlled, we suggest that a differentiated sticker recommendation strategy is needed depending on personality.

The implications of this study are as follows. First, although the model is complex, the individual differences related to the purchase of stickers were examined in detail by examining personality factors, motivation, and the TAM, showing that different personality traits are associated with different motivations for using stickers, and these motivations influence the purchase of stickers. In consumer research, providing personalized products is of significant economic benefit and promotes consumer satisfaction. In the case of selling products offline, it is virtually impossible to personalize them individually, but in an environment that sells stickers online, it is relatively easy to provide recommended stickers tailored to individuals. For example, after discerning which motivation is more closely related to each sticker, stickers can be recommended to express emotion or represent greater intimacy to a person with high agreeableness. Jeong et al. (2020) created a tourism recommendation system suited to a user’s personality type. Similarly, stickers can also be marketed using deep learning to provide an adaptive recommendation system tailored to users’ personalities. How can we measure a person’s personality? Evans (2017) suggests that it is possible to check people’s personalities online by answering a simple 10-question questionnaire. However, this cannot be forced, so data will only be obtained from those willing to participate in the survey. Many studies predict personality through the way people write mails, use apps, and perform social media activities (Shen et al., 2013; Wang et al., 2015; Tandera et al., 2017; Peltonen et al., 2020). Through the data collected from users who have agreed to provide information, service providers can predict their personalities and recommend suitable stickers.

Second, the sticker market has not yet become active in most personal messengers or SNSs. Accordingly, there have been many studies on the use of stickers, but few studies have been conducted regarding their purchase. This study conducted research based on KakaoTalk, where the sticker market is active, and it is expected that it will be the cornerstone of various purchase-related studies if the sticker market is created in other messengers in the future.

## Conclusion

This study explored the relationship between individual personality, sticker use motivation, and the TAM. Despite its implications, this study has several limitations. First, the participants were recruited from a university. Since most participants were college students in their 20s, it is not easy to generalize the results to all ages. Depending on the occupation group, personal messengers may be used more for work, so future research needs to be conducted. Second, various cultural characteristics were not considered. Personality types appear similar regardless of Western or Eastern culture, but in terms of motivation for using stickers, there may be differences. Because relationships are important in Eastern cultures, the motivation for using stickers is focused on relationships. If another study were to be conducted in Western cultures, we might find other motivations for using stickers. Third, the motivation and TAM variables are correlated. In this study, we built model relationships in which personality affects motivation and motivation affects the TAM. However, people who buy a lot of stickers can find them more useful and enjoyable to justify their purchase actions. Of course, it is not easy for motivation to affect personality. Furthermore, a prior studies examined similar models as this study; most TAM studies are models in which perceived usefulness and enjoyment affect purchase intentions. However, in the future, research should be conducted to develop a better model that takes correlations into consideration. In particular, it would be necessary to use a longitudinal design, or an experiment should be conducted based on more studies that could explain the causal relationship between variables.

This study explored sticker purchase where there was no connection point theoretically. In particular, in previous studies, motivation factors were organized by the researcher’s intuitive judgment or interview summary, whereas in this study, the motivations for using stickers were clearly divided into four factors through factor analysis. This is expected to be of great help in future studies that use motivational factors. Of course, revealing more motivation factors through various studies will be one of the directions for future research.

This study has several practical implications. Currently, a sticker recommendation system is being used. If a recommendation system that considers individual characteristics and motivational factors is applied, more personalized services can be provided. Xu et al. (2016) suggested that personality factors had a significant impact on mobile app adoption, and they developed a machine-learning model that automatically determined users’ personalities based on their installed apps, which can be used to promote apps suited to the user. In addition, Matz et al. (2017) showed that psychological targeting is effective for advertising and digital mass persuasion. If we could identify users’ personalities, it would be possible to recommend the stickers that they are likely to prefer. In future research, it will be possible to develop a deep learning model that can infer personality through the type and use of stickers. Using this model, it is possible to continuously monitor people’s sticker usage and purchase behavior and to provide an optimized recommendation service in consideration of individual characteristics. If services including personalization strategies are presented in the sticker market, they will be a way to ensure both market revitalization and consumer satisfaction.

## Data Availability Statement

The original contributions presented in the study are included in the article/Supplementary Material, further inquiries can be directed to the corresponding author.

## Ethics Statement

The studies involving human participants were reviewed and approved by Public Institutional Review Board in South Korea. Written informed consent for participation was not required for this study in accordance with the national legislation and the institutional requirements.

## Author Contributions

HK was the first person to provide the idea of this study and wrote the first paper. YP analyzed the data and conducted two surveys to rewrite the paper. YS compiled prior studies to derive specific hypotheses and also conducted analysis. HC was responsible for the survey, organizing data, coding data, and creating figures and tables. SK provided continuous feedback on the entire research and provided an environment for full research. All authors contributed to the article and approved the submitted version.

## Conflict of Interest

HK and SK were employed by company Able Edutech.

The remaining authors declare that the research was conducted in the absence of any commercial or financial relationships that could be construed as a potential conflict of interest.

## Publisher’s Note

All claims expressed in this article are solely those of the authors and do not necessarily represent those of their affiliated organizations, or those of the publisher, the editors and the reviewers. Any product that may be evaluated in this article, or claim that may be made by its manufacturer, is not guaranteed or endorsed by the publisher.
